# Evaluation of Gingival Microleakage in Class II Composite Restorations with Different Lining Techniques: An In Vitro Study

**DOI:** 10.1155/2015/896507

**Published:** 2015-11-29

**Authors:** Vedavathi Bore Gowda, B. V. Sreenivasa Murthy, Swaroop Hegde, Swapna Devarasanahalli Venkataramanaswamy, Veena Suresh Pai, Rashmi Krishna

**Affiliations:** ^1^Department of Conservative Dentistry and Endodontics, Dayananda Sagar College of Dental Sciences, Bangalore 560 078, India; ^2^Department of Conservative Dentistry and Endodontics, M.S. Ramaiah Dental College, Karnataka 560054, India

## Abstract

*Aim*. To compare the microleakage in class II composite restorations without a liner/with resin modified glass ionomer and flowable composite liner.* Method*. Forty standardized MO cavities were prepared on human permanent mandibular molars extracted for periodontal reasons and then divided into 4 groups of ten specimens. The cavity preparations were etched, rinsed, blot dried, and light cured and Adper Single Bond 2 is applied. Group 1 is restored with Filtek P60 packable composite in 2 mm oblique increments. Group 2 is precure group where 1 mm Filtek Z350 flowable liner is applied and light cured for 20 sec. Group 3 is the same as Group 2, but the liner was cocured with packable composite. In Group 4, 1 mm RMGIC, Fuji Lining LC is applied and cured for 20 sec. All the teeth were restored as in Group 1. The specimens were coated with nail varnish leaving 1 mm around the restoration, subjected to thermocycling, basic fuchsin dye penetration, sectioned mesiodistally, and observed under a stereomicroscope.* Results*. The mean leakage scores of the individual study groups were Group 1 (33.40), Group 2 (7.85), Group 3 (16.40), and Group 4 (24.35). Group 1 without a liner showed maximum leakage. Flowable composite liner precured was the best.

## 1. Introduction

Composite resins are being widely used these days for restoring posterior teeth due to advances in the material aspect and adhesive resin technology. A major disadvantage with the use of conventional composite resin is high polymerization shrinkage. This results in microleakage, staining at the margins of the restoration, recurrent caries, post-op sensitivity, and development of pulpal and periapical pathology [1, 2].

To overcome this, condensable/packable composites were introduced which could be handled like amalgam. They showed less polymerization shrinkage and could be bulk cured [3, 4]. However, adapting these stiffer materials to internal cavity walls and cavosurface margins was difficult [3]. So attempts were made to offset this problem by placing materials with low viscosity as liners under these packable composites which adapted to the cavity better, thus reducing the microleakage.

So, the purpose of this in vitro study was to evaluate gingival microleakage of posterior packable composite resin restorations with and without a liner using open sandwich technique in a class II preparation.

## 2. Materials and Methodology

40 freshly extracted noncarious permanent human mandibular molars were obtained after ethical clearance from M.S. Ramaiah institutions ethical committee, cleaned of surface debris with an ultrasonic scaler and stored in saline at room temperature.

### 2.1. Cavity Preparation

Class II mesioocclusal cavities were prepared using straight and pear shaped diamond points. Dimensions of the prepared cavity were 2 mm wide buccolingually and 2 mm deep pulpally. Gingival seat of the proximal box was placed 1 mm above cementoenamel junction and was 1.5 mm wide. The teeth were randomly divided into 4 groups of 10 teeth in each group.

### 2.2. Materials Used

The following materials have been used:

 Scotchbond multipurpose etchant, 37% phosphoric acid, 3M ESPE;
 bonding agent, Adper Single Bond 2, 3M ESPE;
 flowable composite, Filtek Z 350, 3M ESPE;
 packable composite, Filtek P60, 3M ESPE;
 cavity conditioner, 20% polyacrylic acid;
 GC Fuji Lining LC Paste Pak, GC;
 modelling wax;
 nail varnish;
 2% basic fuchsin dye solution;
 normal saline.

### 2.3. Restorative Procedure

Scotchbond multipurpose etchant was applied to the entire prepared cavity of groups 1, 2, and 3 for 15 sec, rinsed with water for 10 seconds, blot dried, gently air thinned, and light cured for 10 seconds using Spectrum 800 light curing unit from Dentsply and 2-3 consecutive coats of Adper Single Bond 2 Adhesive were applied.

The specimens were then restored as follows:

 Group 1 is restored with Filtek P60-packable composite in less than 2 mm oblique increments and cured for 40 seconds.
 Group 2 is precure group, where 1 mm flowable composite liner is applied to pulpal floor, axial wall, and gingival seat and light cured for 20 seconds.
 Group 3 is cocure group, where 1 mm of flowable composite liner is applied and subsequent 2 mm packable composite increment is placed, and all were cured together for 40 seconds.
 Group 4 is GC liner group where cavity preparation was preconditioned with 20% polyacrylic acid for 15 seconds and then 1 mm GC Fuji Lining LC Paste is applied as a liner and light cured for 20 seconds.

Remainder of the cavities in Groups 2, 3, and 4 were restored as in Group 1.

### 2.4. Evaluation of Microleakage

The specimens were subjected to thermocycling for 500 cycles between 5 ± 2°C and 55 ± 2°C with a dwell time of 30 seconds in each bath and 20 sec interval between baths at ambient air. Root apices of teeth samples were sealed with modeling wax and then painted with 2 coats of nail varnish within 1 mm of restoration margin and then soaked in 2% basic fuchsin dye for 24 hours. The teeth were sectioned mesiodistally into two halves in a vertical plane parallel to long axis of the tooth using diamond disc at slow speed with water spray. The sectioned specimens were mounted on slides and the degree of dye penetration was evaluated under a stereomicroscope.

#### 2.4.1. Scoring Criteria for Assessing Dye Leakage at the Gingival Margin: Leevaloj C, Cochran MA et al

**Figure 1:**
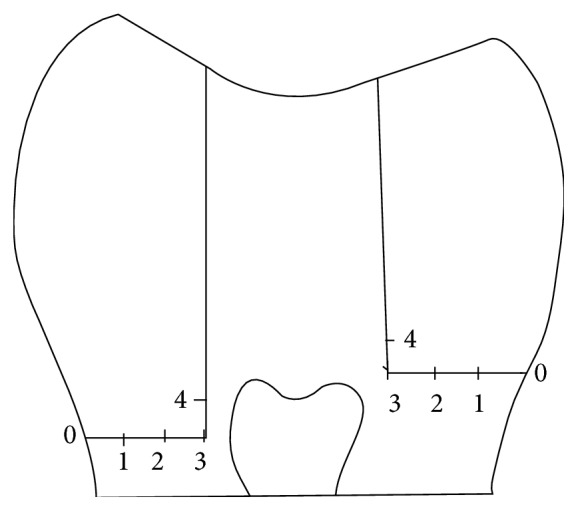


The scoring criteria are as follows (see Figure 1):

 degree 0: no dye penetration,
 degree 1: up to 1/2 the gingival seat,
 degree 2: >1/2 the gingival seat,
 degree 3: all along the gingival seat,
 degree 4: degree 3 plus into the axial wall.

### 2.5. Results

**Figure 2:**
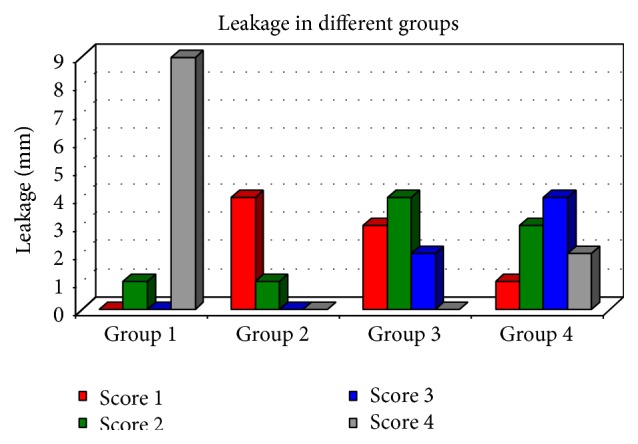
Frequency of microleakage scores in different groups.

**Figure 3:**
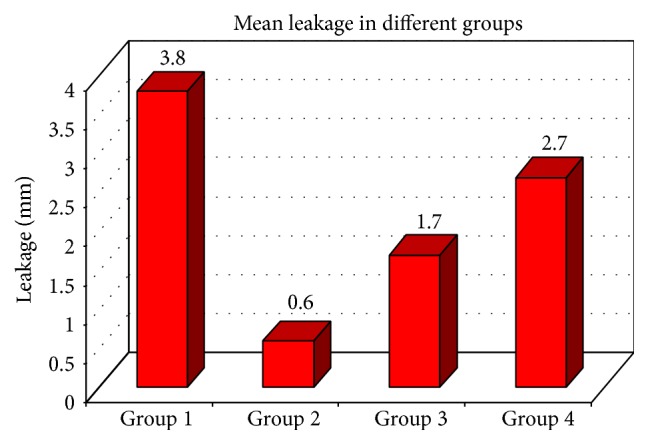
Comparison of mean of microleakage scores in different groups.

See Tables 1–3 and Figures 2 and 3.

### 2.6. Statistical Analysis

The following methods of statistical analysis have been used in this in vitro study. Kruskal-Wallis test was used to compare the mean values of 4 different groups and Mann-Whitney *U* test was used to compare the different groups with each other and find out which groups differ significantly from the other groups.

## 3. Results

Following the microleakage scores (Table 1, Figure 2) and statistical analysis of the values obtained the following observations were made:

- Group 1 specimens without any liner showed maximum microleakage (mean: 33.40) amongst all the experimental groups and this difference was statistically significant.
- Amongst the liner groups, Group 2 specimens showed least leakage values compared to the other groups with liner (Groups 3 and 4).
- Group 4 specimens showed most leakage (mean: 24.35) as compared to the other two liner groups.

**Figure 4 fig4:**
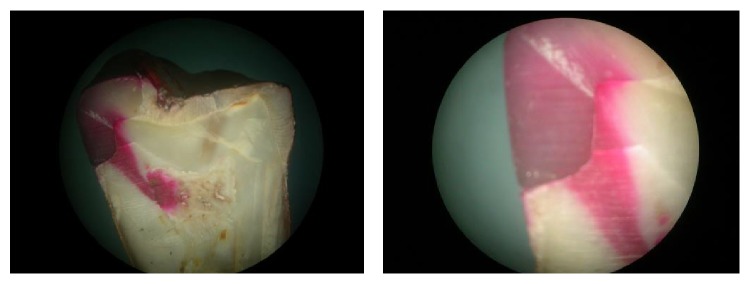
Group 1: microleakage seen under composite restorations without any liner.

**Figure 5 fig5:**
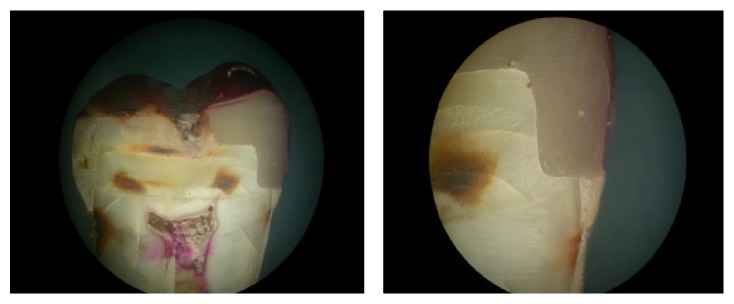
Group 2: microleakage seen under composite restorations with flowable composite liner, precure group.

**Figure 6 fig6:**
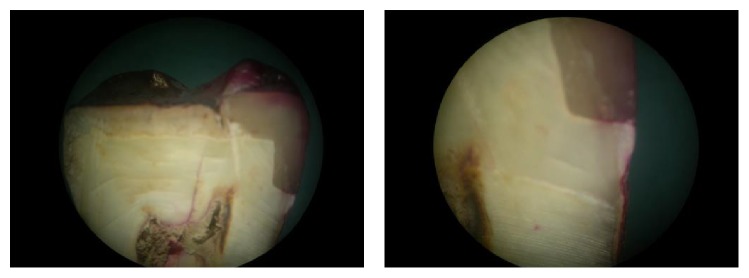
Group 3: microleakage seen under composite restorations with flowable composite liner, cocure group.

**Figure 7:**
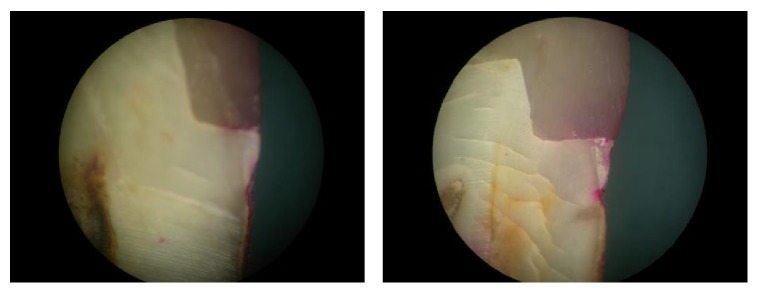
Group 4: microleakage seen under composite restorations with RMGIC liner.

The mean leakage scores of the individual study groups were Group 1 (33.40), Group 2 (7.85), Group 3 (16.40), and Group 4 (24.35). The mean difference observed was statistically significant (*p* < 0.05) between the individual groups (Table 2, Figures 3 [Fig fig4] [Fig fig5] [Fig fig6]–7).

Further analysis by Mann-Whitney *U* test revealed that the mean difference between Group 1 versus Group 2 was <0.001; Group 1 versus Group 3 was <0.001; Group 1 versus Group 4 was <0.005; Group 2 versus Group 3 was <0.013; Group 2 versus Group 4 was <0.001; Group 3 versus Group 4 was <0.043. The *p* value was less than 0.05 indicating that the difference between the groups was statistically significant (Table 3).

## 4. Discussion

Packable composites are indicated for posterior stress bearing areas due to their less polymerization shrinkage and improved handling properties, with an application technique similar to amalgam. Although packable resins do not stick to dental instruments, they were difficult to adapt to cavity preparation due to their stiffness. So application of a cavity liner in areas of difficult access or flow was thought to reduce microleakage [5]. A cavity liner acts as a stress breaker, reduces C-factor, has good flow due to low viscosity, and decreases the bulk of the overlying packable composite. Flowable composites shrink more because they have less filler loading, so they were applied as a thin liner of 1 mm thickness to minimize the effect [6].

In our study, we have placed composite resin in less than 2 mm oblique increments. The rationale is that minimal shrinkage takes place in each increment and the C-factor is reduced due to large free surface that permits resin to flow during polymerization providing a better sealing of the gingival increment [7, 8].

In the present study, specimens with flowable composite liner showed statistically less microleakage as compared to RMGI liner group [3, 9] and this could be due to

1. good adaptation of flowable composite to the prepared tooth structure providing an intimate union with the microstructural defects of the cavity preparation,
2. flowable composite with a low modulus of elasticity and/or low surface tension and increased flexibility that would have ameliorated the stresses of polymerization shrinkage and preserved the integrity of bond to tooth structure [10],
3. the fact that there is minimal internal porosities incorporated within the material.

Current flowable materials can be easily syringed into the cavity but are sometimes difficult to manipulate because of their stickiness. Air gets trapped in the restoration while removing the syringe tip from the cavity. So care must be taken to apply the material in one direction with a gentle releasing motion [11].

It was thought that cocuring the flowable liner and the overlying composite together would help the uncured liner to penetrate better and improve sealing at the margin due to hydraulic pressure of overlying heavier viscosity composite [11]. Contrasting the above finding, our study showed more leakage with CO-CURE compared to PRE-CURE liner group. This may be attributed to the fact that polymerization shrinkage of overlying packable composite would have created contraction forces that may have disrupted the bond of uncured flowable composite liner from the cavity walls. On the other hand, many composites are sticky and have a tendency to pull back as the instruments used to place them are being removed. Also there is an increase in polymerization stresses created due to large volume of polymerizing material [9]; also flowable composite liner if cured serves as a well-adapted first increment, resists disturbance, and absorbs polymerization shrinkage of the overlying composite [4, 12, 13].

RMGIC can be photocured, showed early resistance to moisture contamination, is easy to place, sets on command, and bonds chemically to composite resin. A 41% reduction in the volumetric contraction of resin composite restorations lined with RMGIC has been reported [10]. Extension of conventional GIC to the external cavosurface margin resulted in severe degradation. However, it is now possible to extend RMGIC to external cavosurface (open sandwich) compared to maintaining it short of the margin (closed sandwich) [14].

The reasons for more microleakage seen in specimens lined with RMGIC could be due to the following [10]:

1. Resin component of Fuji II LC would have undergone different rates of polymerization shrinkage during light curing, leading to gap formation at tooth restoration interphase.
2. Resin framework of Fuji II LC may be more rigid and less capable of elastic deformation leading to disruption of bond at the tooth restoration interphase during initial curing.
3. Particle size and viscosity of RMGIC are more comparable to flowable composite.
4. RMGIC is a two-component system, so there are more chances of porosities.
5. RMGIC may be sensitive to dehydration leading to severe loss of water, resulting in considerable changes in the form of failure at the tooth restoration interface.

In our study, we have subjected the specimens to thermocycling to simulate the clinical setting. The scoring criterion used in this dye penetration study is the same as that followed in previous studies [15].

The results of our study showed that there was statistically significant reduction in gingival microleakage in the study groups when a liner was placed under packable composite resin in comparison to the group without any liner.

## 5. Conclusion

Within the limitations of the present in vitro study, it can be concluded that precured flowable composite liner is more effective in sealing the gingival cavosurface margins of class II preparation compared to other groups.

## Figures and Tables

**Table 1:** Frequency of microleakage scores.

	Group 1	Group 2	Group 3	Group 4
Score 1	0	4	3	1
Score 2	1	1	4	3
Score 3	0	0	2	4
Score 4	9	0	0	2

**Table 2:** Mean of dye penetration scores in different groups using Kruskal-Wallis test.

Ranks			
	Group	*N*	Mean rank
Dye penetration scores	Group 1	10	33.40
	Group 2	10	7.85
	Group 3	10	16.40
	Group 4	10	24.35
	Total	40	

**Table 3:** Comparison of leakage in different groups using Mann-Whitney test.

Group	Mean rank	*p* value
1	15.45	<0.001^*∗*^
2	5.55	

1	15.10	<0.001^*∗*^
3	5.90	

1	13.85	0.005^*∗*^
4	7.15	

2	7.35	0.013^*∗*^
3	13.65	

2	5.95	<0.001^*∗*^
4	15.05	

3	7.85	0.043^*∗*^
4	13.15	

^*∗*^The difference between the groups is statistically significant.

## Clinical Significance

There was statistically significant reduction in gingival microleakage when a liner was placed under packable composite resin in comparison to the groups without any liner. It should be noted that these results are based on in vitro data; therefore, further in vivo studies that evaluate the clinical performance of flowable composite and RMGI as liners under packable composites are necessary.
